# Real-world safety of PCSK9 inhibitors: A pharmacovigilance study based on spontaneous reports in FAERS

**DOI:** 10.3389/fphar.2022.894685

**Published:** 2022-11-24

**Authors:** Zhen Feng, Xiaoye Li, Wai Kei Tong, Qingfeng He, Xiao Zhu, Xiaoqiang Xiang, Zhijia Tang

**Affiliations:** ^1^ Department of Clinical Pharmacy and Pharmacy Administration, School of Pharmacy, Fudan University, Shanghai, China; ^2^ Department of Pharmacy, Zhongshan Hospital, Fudan University, Shanghai, China

**Keywords:** alirocumab, evolocumab, PCSK9 inhibitors, FAERS, pharmacovigilance, drug safety, age-and gender-tailored treatment

## Abstract

**Objective:** We aimed to evaluate alirocumab- and evolocumab-related adverse events (AEs) in real-world compared with all other drugs, overall and by gender and age subgroups; we also aimed to compare their risks of cognitive impairment, musculoskeletal disorders and diabetes with various statins and ezetimibe.

**Methods:** We retrospectively extracted AE reports from the FDA Adverse Event Reporting System (FAERS) database during July 2015-June 2021. Disproportionality analyses were performed using reporting odds ratios (RORs) to detect AE signals of alirocumab and evolocumab in the overall population and in different age and gender subgroups, respectively.

**Results:** Compared with all other drugs, both alirocumab and evolocumab had a significant signal in “musculoskeletal and connective tissue disorders” (ROR_1_ = 2.626, 95% CI 2.552–2.702; ROR2 = 2.575, 95% CI 2.538–2.613). The highest ROR value of 2.311 (95% CI 2.272–2.351) was for “injury, poisoning and procedural complications” and was found in patients aged ≥65 years on evolocumab. The most frequent AEs were “general disorders and administration site conditions” and “musculoskeletal and connective tissue disorders” for all subpopulations. At the preferred term level, the most frequent AE signal was myalgia for alirocumab and injection site pain for evolocumab, overall and by subgroups. Compared with statins/ezetimibe, PCSK9 inhibitors exhibited lower ROR values for adverse events associated with SOC “nervous system disorders”, “psychiatric disorders” and “metabolism and nutrition disorders” (all RORs < 1), but mixed results for musculoskeletal disorders. Compared with all other drugs, undocumented AEs, such as acute cardiac event (ROR = 30.0, 95% CI 9.4–95.3) and xanthoma (ROR = 9.3, 95% CI 3.4–25.5), were also reported.

**Conclusion:** Real-world evidence showed that PCSK9 inhibitors were associated with an increased risk of musculoskeletal and connective tissue disorders and general disorders and administration site conditions, overall and by subgroups. Muscle toxicity, injection site reactions, and influenza-like illness were significant AE signals. Compared with various statins and ezetimibe, PCSK9 inhibitors have shown a favorable safety profile in muscle-related events, cognitive impairment and diabetes. Some undocumented AE signals were also reported. Due to the limitations of spontaneous reporting databases, further studies are still needed to establish causality and validate our results.

## Introduction

Hyperlipidemia is a major risk factor for atherosclerotic cardiovascular disease (ASCVD), which remains the leading cause of morbidity and mortality worldwide. Low-density lipoprotein cholesterol (LDL-C) is known to be a major contributor to ASCVD. Typically, statins are the first-line therapy. In addition to statins, a new class of drugs called proprotein convertase subtilisin/kexin type 9 (PCSK9) inhibitors has been approved by the U.S. Food and Drug Administration (FDA) and the European Medicines Agency (EMA) in 2015 for the treatment of primary hyperlipidemia and familial hypercholesterolemia (HeFH). In randomized controlled trials (RCTs), two PCSK9 inhibitors, alirocumab and evolocumab, have shown encouraging results in preventing major vascular events in high-risk ASCVD patients compared to placebo (Giugliano et al., 2017b; Sabatine et al., 2017; Schwartz et al., 2018; Szarek et al., 2019). They can also decrease low-density lipoprotein cholesterol (LDL-C) levels by approximately 60%, even in those already receiving the maximum dose of statins, and therefore have emerged as one of the important therapies for ASCVD patients (Sabatine, 2019).

While evidence from clinical trials indicated that PCSK9 inhibitors seem to be well tolerated, real-world evidence on age- and gender-related differences is scarce, especially for some rare but severe adverse events (AEs). Myotoxicity is seen as one of the growing concerns in gaining optimal patient compliance with statins (Pirillo and Catapano, 2015). Although the underlying mechanism remains unclear, it may be related to novel immunogenetic factors, gender, and more (Nikolic et al., 2020). In theory, PCSK9 inhibitors are fully human monoclonal antibodies, and this characteristic may reduce the risk of immunogenicity. The mechanism of myotoxicity of PCSK9 inhibitors needs further study. While RCTs demonstrated no difference in the occurrence of myalgia between patients receiving PCSK9 inhibitors and placebo (OR = 0.95, *p* = 0.65) (Karatasakis et al., 2017; Sabatine et al., 2017; Schwartz et al., 2018), a real-world study regarded it as a major reason for treatment interruption (Gurgoze et al., 2019). Just as musculoskeletal disorders are more common and more likely to induce drug discontinuation in women over 65 than in younger men (Hopewell et al., 2012; Karalis et al., 2016; Cangemi et al., 2017), it is urgent to clarify whether specific high-risk subgroups exist for PCSK9 inhibitors.

Some preclinical studies also suggested that low cholesterol levels in the brain may cause cognitive impairment (Rojas-Fernandez et al., 2014) as 25% cholesterol is present in myelin (Bjorkhem and Meaney, 2004). The potentially harmful effects of the extremely low LDL-C levels induced by statins and PCSK9 inhibitors on cognitive function have attracted attention (Rojas-Fernandez et al., 2014). To date, although the FDA has warned about the potential neurocognitive risk of evolocumab (Smith, 2014), there is no conclusive evidence from clinical trials fully establishing the relationship (Benn et al., 2017; Ridker et al., 2017; Lyall et al., 2018; Yuet et al., 2021), but intensive monitoring was recommended in clinical practice for those who were treated for more than 3 years, over 75 years of age, or at very high ASCVD risk as these subgroups of patients were underrepresented in trials (Kosmas et al., 2020). Since age and gender have been introduced as the most prominent variables in assessing cognitive impairment (Podcasy and Epperson, 2016), we need compelling data to support any age- and gender-tailored recommendation for PCSK9 inhibitors.

Diabetes is another commonly suspected side effect of lipid-lowering agents (Sattar et al., 2010; Adhyaru and Jacobson, 2018). FDA has changed the labeling of all statins to emphasize the diabetogenic effects, especially at high-intensity doses, to increase hemoglobin A1c and/or fasting plasma glucose (Ray, 2013). In mice models, PCSK9 deficiency was shown to reduce insulin secretion and induce glucose intolerance due to toxic cholesterol accumulation within *β* cells (Da Dalt et al., 2019). Blood glucose levels were also slightly elevated at 52 weeks in the SPIRE-1 and SPIRE-2 trials of bococizumab, although the increase in newly diagnosed diabetes was not significant (Ridker et al., 2017). The association between single nucleotide polymorphisms in PCSK9 and the incidence and prevalence of type 2 diabetes was partially confirmed by a Mendelian randomization study (OR_1_ = 1.15, 95% CI 0.76–1.72; OR_2_ = 1.26, 95% CI 0.88–1.80) (Benn et al., 2017). Given the higher prevalence of diabetes among men and the elderly, the question arises if they also have a greater risk of glucose abnormalities when using PCSK9 inhibitors.

As mentioned above, details of the adverse effects of PCSK9 inhibitors in subpopulations are unclear. Evidence from real-world data is urgently required to verify these findings and provide recommendations for clinicians on the rational use of medications. This study evaluated the comprehensive AE signals of PCSK9 inhibitors using the FDA Adverse Event Reporting System (FAERS) database, overall and in age- and gender-oriented perspectives. In addition, we compared their AE signals of interest (including cognitive impairment, musculoskeletal disorders, and diabetes) with those of various statins and ezetimibe.

In recent years, FAERS and other pharmacovigilance databases, such as the European Pharmacovigilance Database (Eudra Vigilance), Japanese Adverse Drug Event Report Database (JADER) and WHO Vigibase, have played important roles in detecting and identifying new, rare and serious adverse drug reactions and events (Lopes et al., 2013; Pal et al., 2013; Inacio et al., 2017). The FAERS database contains real-world AE reports from large populations that may be overlooked in well-designed clinical trials and have become an immensely valuable resource to support post-marketing surveillance and early detection of drug safety issues (Inacio et al., 2017; Cirmi et al., 2020). Reports in the FAERS database are submitted by healthcare professionals, consumers and manufacturers spontaneously. The database was updated quarterly and can be downloaded publicly on the FDA website. Since 1968, it has received over 24 million safety reports. Therefore, FAERS is a useful tool for finding safety issues that might be related to PCSK9 inhibitors.

## Materials and methods

### Data source and collection

The FAERS database contains adverse reports, medication error reports, and product quality complaints, which are used for post-marketing safety surveillance of drugs.

We conducted a retrospective search in the FAERS database for AE reports related to PCSK9 inhibitors from July 2015 to June 2021. First, we filtered out all reports associated with PCSK9 inhibitors by searching for the trade and generic drug names (i.e., alirocumab, praluent, evolocumab, repatha, and pack9), as well as common spelling errors (e.g., “simvastin” for simvastatin). Reports related to seven statins (atorvastatin, fluvastatin, lovastatin, pitavastatin, pravastatin, rosuvastatin, and simvastatin) and ezetimibe were also extracted during the same period. Only reports that identified the drug as primary suspect were retained. As recommended by the FDA, if multiple reports of the same event were detected, only the most recent case version of each event was retained. We further excluded suspected duplicate reports. Then, additional information for each report was collected, including demographic and administrative information details (i.e., patient’s age, gender, AE occurrence date, reporting year, reporter’s occupation, reporting country) and drug information details (i.e., drug name, administration route).

All AEs in the FAERS were coded using preferred terms (PT) and primary system organ class (SOC) according to Medical Dictionary for Regulatory Activities (MedDRA) (English version 24.0) (Thiessard et al., 2005; de Langen et al., 2008; Sarntivijai et al., 2016; Wu et al., 2019). We selected four MedDRA SOCs of interest (“nervous system disorders”, “psychiatric disorders”, “metabolism and nutrition disorders”, and “musculoskeletal and connective tissue disorders”) for risk comparison with statins and ezetimibe. The adverse events unrelated to the drug itself were excluded, including “product issues”, and “social circumstances”. These subcategories were based on the MedDRA hierarchy and were manually validated by two researchers.

### Outcomes

The primary outcome was any AE signals associated with alirocumab and evolocumab compared to all other drugs in the overall population at the system organ class and preferred term level, and by age/gender subgroups. Secondary outcomes were AEs of special interest compared to various statins and ezetimibe (including “nervous system disorders”, “psychiatric disorders”, “metabolism and nutrition disorders”, and “musculoskeletal and connective tissue disorders”).

### Statistical analysis

The descriptive analysis was performed to summarize characteristic profiles of AE reports associated with PCSK9 inhibitors (i.e., gender, age, reporting year, reporting country, and type of reporter). Continuous data were expressed as means (standard deviation, SD) or medians (interquartile range, IQR). Categorical variables were described as frequencies and percentages.

We conducted a disproportionality analysis to detect AE signals, based on the 2 × 2 contingency table (Supplementary Table S1) (Zink et al., 2013). Its principle is to compare the difference between the frequency of the target drug event and the background frequency. This study calculated reporting odds ratio (ROR) value and its 95% confidence interval (CI) and proportional reporting ratio (PRR) value and its χ^2^ value to detect AE signals of alirocumab and evolocumab, respectively. A higher ROR value indicates a higher probability of AEs (Sakaeda et al., 2013). The standard threshold for a signal was determined as: the number of AE reports ≥3, the lower bound of 95% CI > 1, ROR >2.0, PRR >2.0 and χ^2^ > 4 (van Puijenbroek et al., 2002; Sakaeda et al., 2013). To further assess the safety of subgroups, we grouped subjects according to demographic characteristics such as age and gender and analyzed the data separately. We also performed comparisons within subgroups (i.e., female vs. male, <65 years vs. ≥ 65 years). To further assess the safety of muscular disorders, cognitive impairment, and diabetes, we performed the same disproportionality analysis with statins/ezetimibe as the control group. Data processing and statistical analysis were performed using R Studio (version 1.4.1717, PBC, United States).

## Results

**FIGURE 1:**
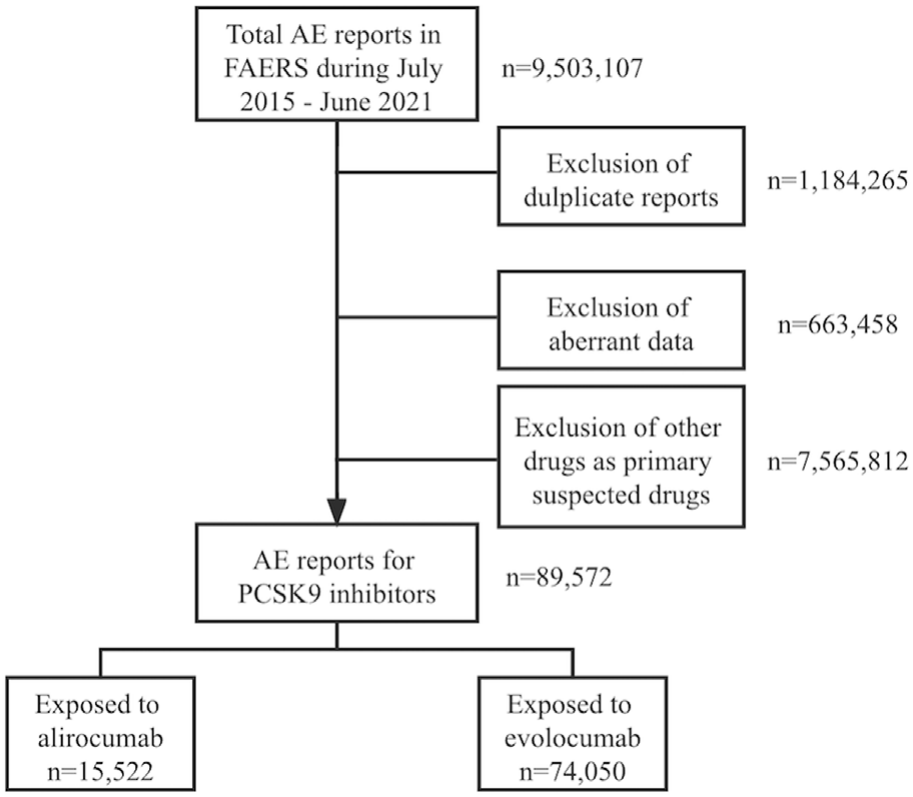
Flowchart of data collection process.

In total, 7,655,384 AE reports were obtained from the FAERS database from July 2015 to June 2021 after excluding duplicates and incomplete reports (i.e., cases lacking AE date, gender, and age at the same time). Aberrant data (i.e., AE occurrence date before the date of drug use, or missing drug names) were also excluded from subsequent analysis. Finally, 15,522 and 74,050 reports were identified, with alirocumab and evolocumab as the primary suspected drug, respectively (Figure 1).

### Characteristics of AE reports

As shown in Table 1, among all 89,572 AE reports, women had a higher reporting rate than men (alirocumab: 55.2%; evolocumab: 56.6%). The median age of cases was 68.0 (61.0–74.0) years and 67.0 (60.0–74.0) years, respectively. The number of cases peaked in 2018 (evolocumab: 43,419, 58.6%) and 2019 (alirocumab: 4,796, 30.9%).

**TABLE 1:** Characteristics of AE reports associated with PCSK9 inhibitors from July 2015 to June 2021.

Characteristics		Alirocumab (*n* = 15,522)	Evolocumab (*n* = 74,050)
Sex (n, %)	Male	6,057 (39.0)	31,418 (42.4)
	Female	8,575 (55.2)	41,936 (56.6)
	Unknown	890 (5.7)	696 (0.9)
Years of age, (median, IQR)	All	68 (61–74)	67 (60–74)
	Male	67 (60–73)	66 (59–73)
	Female	69 (62–75)	68 (61–75)
Reporting year (n, %)	2015 (July-December)	211 (1.4)	600 (0.8)
	2016	2,247 (14.5)	4,024 (5.4)
	2017	2,718 (17.5)	5,146 (6.9)
	2018	3,519 (22.7)	43,419 (58.6)
	2019	4,796 (30.9)	8,514 (11.5)
	2020	1,741 (11.2)	8,018 (10.8)
	2021 (January-June)	290 (1.9)	4,329 (5.8)
Reporting country (n, %)	US	14,943 (96.3)	71,319 (96.3)
	Other/unknown	569 (3.7)	2,731 (3.7)
Type of reporter (n, %)	Consumer	11,309 (72.9)	19,890 (26.9)
	Physician	1,306 (8.4)	41,944 (56.6)
	Other health professional	2,776 (17.9)	11,505 (15.5)
	Other/unknown	131 (0.8)	711 (1.0)

### Comprehensive AE signal analysis overall and by subgroups

After excluding duplicate and aberrant cases, alirocumab and evolocumab reported 41,639 and 165,946 events in 26 and 27 SOCs, with an average of 2.7 and 2.2 events per case, respectively. We compared the number of AE reports for alirocumab and evolocumab at the SOC level with AE reports in the overall population at the same SOC level. After screening, SOCs unrelated to drugs, such as product issues, were excluded. Finally, the results showed that both alirocumab and evolocumab had a significant signal at SOC level in “musculoskeletal and connective tissue disorders” compared with all other drugs (alirocumab: ROR = 2.626, 95% CI 2.552–2.702; evolocumab: ROR = 2.575, 95% CI 2.538–2.613) (Table 2). Based on subgroups analyses (Tables 3,4), for both alirocumab and evolocumab, each gender and age group showed similar but significantly increased risk in “musculoskeletal and connective tissue disorders”. In addition, a signal for “injury, poisoning and procedural complications” was found for evolocumab in each gender and age group, while ≥65 age group presented the highest ROR value (2.311, 95% CI 2.272–2.351). In addition, comparisons within the subgroups showed that males on alirocumab had a higher risk of “congenital, familial and genetic disorders”, and females on evolocumab carried a higher risk of “endocrine disorders”. Compared to patients ≥65 years, patients <65 years were more likely to develop “hepatobiliary disorders” on alirocumab and “congenital, familial and genetic disorders” on evolocumab.

**TABLE 2:** Comparison of the number of AE reports between alirocumab, evolocumab and all other drugs at the system organ class level.

System organ class	No. of AE reports		ROR_1_ (95% CI)	ROR_2_ (95% CI)
	Alirocumab	Evolocumab		
Blood and lymphatic system disorders	94	358	0.163 (0.133–0.200)	0.156 (0.140–0.173)
Cardiac disorders	680	1,881	0.389 (0.361–0.420)	0.268 (0.256–0.280)
Congenital, familial and genetic disorders	11	25	0.229 (0.127–0.414)	0.130 (0.088–0.193)
Ear and labyrinth disorders	193	978	1.383 (1.201–1.594)	1.768 (1.660–1.883)
Endocrine disorders	23	123	0.087 (0.058–0.130)	0.116 (0.097–0.138)
Eye disorders	508	1,604	0.910 (0.834–0.993)	0.718 (0.683–0.754)
Gastrointestinal disorders	2,668	8,912	0.816 (0.785–0.849)	0.676 (0.661–0.690)
General disorders and administration site conditions	10,249	39,706	1.673 (1.636–1.711)	1.615 (1.597–1.634)
Hepatobiliary disorders	105	342	0.396 (0.327–0.480)	0.323 (0.290–0.359)
Immune system disorders	301	1,305	0.261 (0.233–0.293)	0.284 (0.269–0.300)
Infections and infestations	1,781	6,676	1.153 (1.100–1.210)	1.082 (1.056–1.109)
Injury, poisoning and procedural complications	5,025	32,815	1.044 (1.013–1.075)	2.083 (2.058–2.109)
Investigations	2,311	8,527	1.344 (1.289–1.402)	1.231 (1.205–1.259)
Metabolism and nutrition disorders	345	1,309	0.387 (0.348–0.430)	0.367 (0.347–0.387)
Musculoskeletal and connective tissue disorders	5,476	21,343	2.626 (2.552–2.702)	2.575 (2.538–2.613)
Neoplasms benign, malignant and unspecified (incl cysts and polyps)	165	569	0.264 (0.227–0.308)	0.228 (0.210–0.247)
Nervous system disorders	2,709	9,438	0.721 (0.694–0.750)	0.624 (0.611–0.637)
Pregnancy, puerperium and perinatal conditions	0	6	NA	0.006 (0.003–0.013)
Psychiatric disorders	949	3,396	0.406 (0.381–0.433)	0.363 (0.350–0.375)
Renal and urinary disorders	428	998	0.434 (0.394–0.477)	0.252 (0.236–0.268)
Reproductive system and breast disorders	122	294	0.330 (0.277–0.395)	0.199 (0.177–0.223)
Respiratory, thoracic and mediastinal disorders	2,653	11,324	1.072 (1.031–1.115)	1.155 (1.133–1.177)
Skin and subcutaneous tissue disorders	2,571	6,627	0.982 (0.944–1.022)	0.620 (0.605–0.635)
Surgical and medical procedures	554	2,200	1.808 (1.662–1.966)	1.789 (1.715–1.866)
Vascular disorders	476	1,656	0.227 (0.207–0.248)	0.197 (0.188–0.207)

ROR_1_, alirocumab vs. all other drugs; ROR_2_, evolocumab vs. all other drugs.

**TABLE 3:** Comparison of the number of AE reports between alirocumab and all other drugs at the system organ class level, by gender and age groups.

System organ class	ROR (95% CI)			
	Female	Male	<6 years	≥65 years
Blood and lymphatic system disorders	0.150 (0.113–0.197)	0.156 (0.110–0.220)	0.175 (0.120–0.255)	0.164 (0.123–0.219)
Cardiac disorders	0.305 (0.272–0.341)	0.453 (0.402–0.509)	0.363 (0.312–0.422)	0.350 (0.312–0.392)
Congenital, familial and genetic disorders	0.108 (0.035–0.334)	**0.466 (0.233–0.933)**	0.389 (0.162–0.934)	0.168 (0.063–0.447)
Ear and labyrinth disorders	1.394 (1.159–1.677)	1.342 (1.055–1.706)	1.738 (1.362–2.218)	1.152 (0.925–1.435)
Endocrine disorders	0.084 (0.049–0.145)	0.084 (0.042–0.169)	0.084 (0.038–0.188)	0.099 (0.057–0.170)
Eye disorders	0.913 (0.814–1.024)	0.866 (0.745–1.006)	0.928 (0.785–1.097)	0.915 (0.808–1.036)
Gastrointestinal disorders	0.872 (0.83–0.917)	0.769 (0.718–0.822)	0.961 (0.895–1.031)	0.786 (0.742–0.831)
General disorders and administration site conditions	1.815 (1.763–1.867)	1.449 (1.394–1.507)	1.697 (1.625–1.771)	1.647 (1.596–1.700)
Hepatobiliary disorders	0.331 (0.252–0.436)	0.422 (0.309–0.576)	**0.577 (0.425–0.785)**	0.265 (0.190–0.370)
Immune system disorders	0.272 (0.235–0.315)	0.245 (0.202–0.298)	0.259 (0.208–0.323)	0.234 (0.197–0.277)
Infections and infestations	1.142 (1.073–1.216)	1.157 (1.069–1.252)	1.326 (1.217–1.446)	1.085 (1.012–1.162)
Injury, poisoning and procedural complications	1.187 (1.142–1.233)	1.091 (1.038–1.148)	0.971 (0.914–1.033)	1.249 (1.199–1.301)
Investigations	1.188 (1.120–1.259)	1.630 (1.529–1.739)	1.466 (1.356–1.585)	1.208 (1.135–1.286)
Metabolism and nutrition disorders	0.324 (0.278–0.377)	0.414 (0.349–0.491)	0.389 (0.317–0.477)	0.340 (0.290–0.399)
Musculoskeletal and connective tissue disorders	2.538 (2.444–2.636)	2.909 (2.779–3.045)	2.620 (2.480–2.768)	2.655 (2.550–2.763)
Neoplasms benign, malignant and unspecified (incl cysts and polyps)	0.201 (0.16–0.253)	0.310 (0.244–0.392)	0.197 (0.140–0.277)	0.242 (0.193–0.303)
Nervous system disorders	0.686 (0.651–0.723)	0.769 (0.722–0.82)	0.732 (0.680–0.789)	0.714 (0.675–0.755)
Psychiatric disorders	0.370 (0.339–0.405)	0.481 (0.435–0.531)	0.384 (0.338–0.436)	0.433 (0.396–0.473)
Renal and urinary disorders	0.350 (0.305–0.402)	0.456 (0.391–0.533)	0.480 (0.403–0.572)	0.360 (0.311–0.417)
Reproductive system and breast disorders	0.247 (0.189–0.323)	0.432 (0.333–0.561)	0.303 (0.212–0.434)	0.327 (0.254–0.421)
Respiratory, thoracic and mediastinal disorders	1.035 (0.982–1.091)	1.176 (1.104–1.253)	1.121 (1.040–1.208)	1.111 (1.052–1.174)
Skin and subcutaneous tissue disorders	1.035 (0.983–1.089)	0.954 (0.892–1.021)	1.004 (0.930–1.084)	0.962 (0.908–1.018)
Surgical and medical procedures	1.557 (1.384–1.753)	1.869 (1.628–2.146)	1.626 (1.371–1.928)	1.690 (1.495–1.911)
Vascular disorders	0.218 (0.193–0.246)	0.224 (0.192–0.261)	0.217 (0.181–0.259)	0.250 (0.221–0.282)

Bolds indicate groups with significantly higher risk shown in comparisons within subgroups (i.e., female vs. male, <65 years vs. ≥ 65 years). Data are not presented.

**TABLE 4:** Comparison of the number of AE reports between evolocumab and all other drugs at the system organ class level, by gender and age groups.

System organ class	ROR (95% CI)			
	Female	Male	<65 years	≥65 years
Blood and lymphatic system disorders	0.153 (0.133–0.175)	0.163 (0.138–0.191)	0.160 (0.133–0.192)	0.156 (0.134–0.181)
Cardiac disorders	0.231 (0.217–0.246)	0.325 (0.304–0.347)	0.276 (0.254–0.299)	0.275 (0.258–0.294)
Congenital, familial and genetic disorders	0.114 (0.066–0.196)	0.158 (0.09–0.278)	**0.217 (0.126–0.374)**	0.066 (0.030–0.147)
Ear and labyrinth disorders	1.631 (1.498–1.775)	1.980 (1.801–2.176)	1.351 (1.188–1.536)	1.935 (1.773–2.111)
Endocrine disorders	**0.150 (0.123–0.184)**	0.064 (0.044–0.094)	0.136 (0.101–0.182)	0.115 (0.089–0.149)
Eye disorders	0.753 (0.707–0.801)	0.677 (0.625–0.734)	0.670 (0.612–0.734)	0.806 (0.753–0.862)
Gastrointestinal disorders	0.736 (0.717–0.756)	0.589 (0.568–0.611)	0.705 (0.679–0.732)	0.671 (0.651–0.692)
General disorders and administration site conditions	1.692 (1.668–1.717)	1.497 (1.469–1.524)	1.688 (1.655–1.722)	1.466 (1.442–1.491)
Hepatobiliary disorders	0.280 (0.242–0.325)	0.391 (0.336–0.456)	0.363 (0.303–0.434)	0.288 (0.245–0.339)
Immune system disorders	0.312 (0.292–0.334)	0.241 (0.219–0.265)	0.274 (0.249–0.303)	0.275 (0.254–0.298)
Infections and infestations	1.133 (1.098–1.168)	1.013 (0.973–1.055)	1.157 (1.109–1.207)	1.046 (1.009–1.085)
Injury, poisoning and procedural complications	2.021 (1.989–2.053)	2.220 (2.178–2.262)	2.057 (2.012–2.102)	2.311 (2.272–2.351)
Investigations	1.120 (1.087–1.154)	1.421 (1.375–1.468)	1.298 (1.249–1.349)	1.050 (1.015–1.086)
Metabolism and nutrition disorders	0.362 (0.337–0.389)	0.375 (0.345–0.409)	0.383 (0.348–0.421)	0.355 (0.328–0.385)
Musculoskeletal and connective tissue disorders	2.484 (2.437–2.531)	2.687 (2.627–2.748)	2.402 (2.339–2.466)	2.590 (2.536–2.644)
Neoplasms benign, malignant and unspecified (incl cysts and polyps)	0.193 (0.172–0.217)	0.280 (0.249–0.315)	0.158 (0.132–0.188)	0.267 (0.239–0.298)
Nervous system disorders	0.632 (0.615–0.649)	0.613 (0.593–0.634)	0.617 (0.594–0.64)	0.631 (0.613–0.65)
Pregnancy, puerperium and perinatal conditions	0.010 (0.004–0.022)	NA	0.009 (0.003–0.029)	NA
Psychiatric disorders	0.374 (0.358–0.391)	0.345 (0.327–0.365)	0.390 (0.367–0.413)	0.360 (0.343–0.379)
Renal and urinary disorders	0.233 (0.214–0.253)	0.283 (0.258–0.311)	0.246 (0.220–0.275)	0.260 (0.238–0.284)
Reproductive system and breast disorders	0.185 (0.159–0.216)	0.224 (0.189–0.266)	0.223 (0.184–0.271)	0.175 (0.147–0.209)
Respiratory, thoracic and mediastinal disorders	1.150 (1.121–1.178)	1.166 (1.131–1.202)	1.130 (1.092–1.17)	1.184 (1.152–1.217)
Skin and subcutaneous tissue disorders	0.647 (0.627–0.668)	0.581 (0.558–0.605)	0.566 (0.540–0.592)	0.633 (0.611–0.656)
Surgical and medical procedures	1.712 (1.618–1.81)	1.936 (1.815–2.065)	1.665 (1.540–1.801)	1.801 (1.694–1.915)
Vascular disorders	0.205 (0.192–0.218)	0.188 (0.174–0.204)	0.175 (0.159–0.191)	0.215 (0.201–0.230)

Bolds indicate groups with significantly higher risk shown in comparisons within subgroups (i.e., female vs. male, <65 years vs. ≥ 65 years). Data are not presented.

From the perspective of subgroups, the most frequent AE report at the SOC level was “general disorders and administration site conditions”, followed by “musculoskeletal and connective tissue disorders” and “respiratory, thoracic and mediastinal disorders”, which were consistent within all subpopulations except females on alirocumab (Table 5).

**TABLE 5:** Number of AE reports of PCSK9 inhibitors at the system organ class level by subgroups.

System organ class	Alirocumab				Evolocumab			
	Female	Male	<65 years	≥65 years	Female	Male	<65 years	≥65 years
Blood and lymphatic system disorders	50	32	27	47	209	148	115	170
Cardiac disorders	310	282	170	304	967	899	603	917
Congenital, familial and genetic disorders	3	8	5	4	13	12	13	6
Ear and labyrinth disorders	113	67	65	80	539	435	235	510
Endocrine disorders	13	8	6	13	95	27	45	58
Eye disorders	296	173	139	254	1,001	600	468	855
Gastrointestinal disorders	1,649	902	833	1,280	5,752	3,100	2,900	4,212
General disorders and administration site conditions	6,327	3,286	2,780	5,038	24,525	14,875	12,863	17,568
Hepatobiliary disorders	51	40	41	35	177	164	120	145
Immune system disorders	182	101	80	134	853	439	394	602
Infections and infestations	1,025	639	546	835	4,155	2,486	2,227	3,077
Injury, poisoning and procedural complications	2,961	1,694	1,145	2,651	18,933	13,596	10,095	16,834
Investigations	1,194	991	673	1,039	4,615	3,846	2,787	3,466
Metabolism and nutrition disorders	168	132	93	151	769	530	426	602
Musculoskeletal and connective tissue disorders	3,091	2,142	1,468	2,751	12,360	8,818	6,314	10,245
Neoplasms benign, malignant and unspecified (incl cysts and polyps)	73	69	33	75	287	276	123	316
Nervous system disorders	1,501	1,029	737	1,334	5,688	3,679	2,917	4,536
Psychiatric disorders	504	400	241	502	2,083	1,281	1,137	1,603
Renal and urinary disorders	201	161	127	177	549	444	304	489
Reproductive system and breast disorders	53	57	30	60	163	131	103	123
Respiratory, thoracic and mediastinal disorders	1,491	1,035	742	1,364	6,721	4,534	3,475	5,520
Skin and subcutaneous tissue disorders	1,568	895	704	1,253	4,113	2,467	1,897	3,215
Surgical and medical procedures	278	205	134	258	1,245	936	637	1,046
Vascular disorders	266	168	122	260	1,022	626	458	856

At the preferred terms level, the most frequent AE signal was myalgia for alirocumab and injection site pain for evolocumab, which was consistent in all gender and age groups. Besides, influenza like illness was common among males who used alirocumab (*n* = 264) and patients aged <65 years for both alirocumab and evolocumab (*n*_1_ = 221; *n*_2_ = 910) (Supplementary Tables S2, S3). A list of the positive signals was provided in Supplementary Tables S4, S5. The results showed that a total of 140 and 150 suspicious signals were generated for alirocumab and evolocumab, respectively, involving 21 types of SOCs.

### Risk assessment of AE signals of interest and comparison with statins/ezetimibe

**FIGURE 2:**
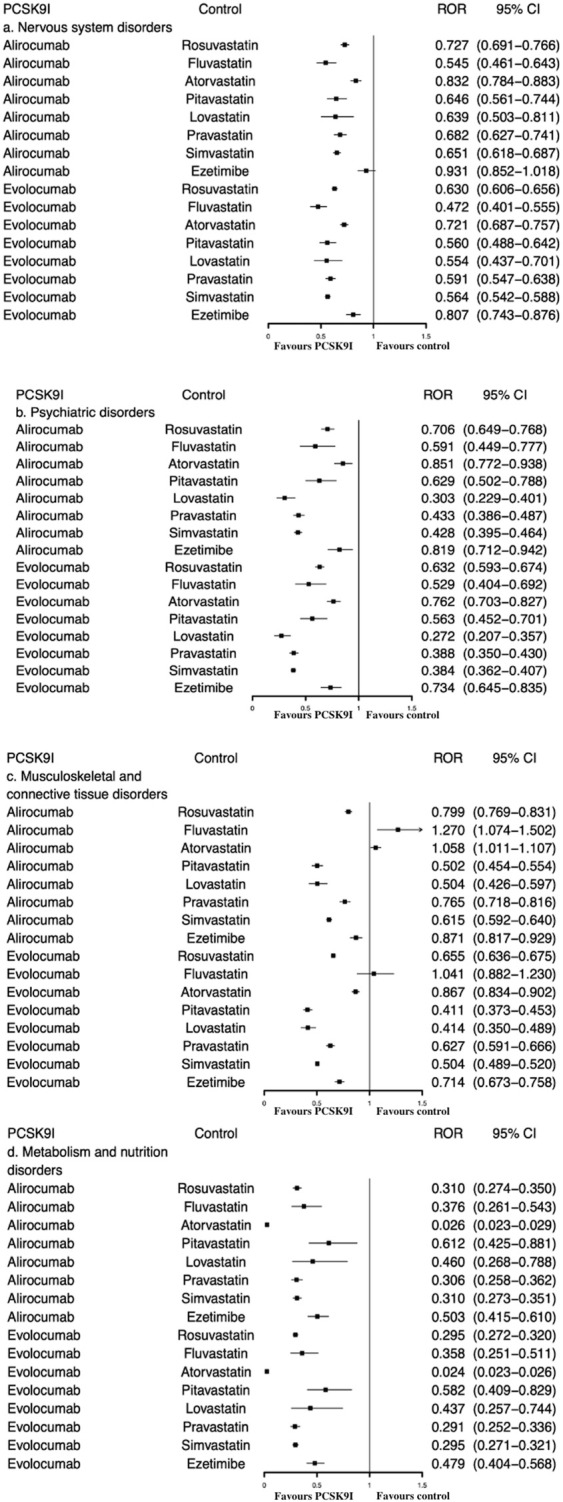
ROR of **(A)** nervous system disorders, **(B)** psychiatric disorders, **(C)** musculoskeletal and connective tissue disorders, and **(D)** metabolism and nutrition disorders between PCSK9 inhibitors and statins/ezetimibe.

As demonstrated by Table 2 and Figure 2, both alirocumab and evolocumab considerably reduced the reporting probability of “nervous system disorders”, “psychiatric disorders” and “metabolism and nutrition disorders”, whether compared with all other drugs or with statins/ezetimibe, overall and by subgroups. Meanwhile, no positive signals associated with “cognitive impairment” were identified, and 35 (0.047%) reports of “glucose tolerance impaired” was only found in evolocumab (ROR = 2.5, 95% CI 1.8–3.5) (Supplementary Tables S4,S5).

When compared with statins/ezetimibe, there was no significant increased signal of “musculoskeletal and connective tissue disorders” associated with PCSK9 inhibitor. Both alirocumab and evolocumab showed lower ROR values (all RORs < 1) (Figure 2).

In addition, compared with all other drugs, we also observed strong AE signals that were not recorded in drug labels, including acute cardiac event (*n* = 3, ROR = 30.0, 95% CI 9.4–95.3) and urinary bladder polyp (*n* = 4, ROR = 20.3, 95% CI 7.5–54.8) caused by alirocumab, and xanthoma caused by evolocumab (*n* = 4, ROR = 9.3, 95% CI 3.4–25.5) (Supplementary Tables S4,S5).

## Discussion

In this study, we quantitatively evaluated the safety profile of PCSK9 inhibitors in real-world settings from an age- and gender-oriented perspective using data from the FAERS database, and compared their risks of cognitive impairment, musculoskeletal disorders and diabetes with various statins and ezetimibe, where PCSK9 inhibitors performed satisfactorily overall.

We included 15,522 and 74,050 AE reports associated with alirocumab and evolocumab. According to the financial reports released by the manufacturers Sanofi and Amgen, as of June 2021, the cumulative sales of alirocumab and evolocumab were 1.165 billion euros and 3.14 billion US dollars, respectively (Amgen, 2021; Sanofi, 2021). The fact that alirocumab’s sales lagged behind evolocumab partly explained why the latter had far more AE reports during the study period.

According to our analysis, the most common AE signals of PCSK9 inhibitors were associated with general disorders and administration site conditions, which was consistent with drug labels and earlier findings (Zhang et al., 2015; Jones et al., 2016; Schwartz et al., 2018). However, the incidence of injection site reactions reported in our study was lower than in the literature (24.9% vs. 33.8%) (Gurgoze et al., 2019). This may be because the voluntary reporting scheme cannot cover all AEs that have occurred in the real world, and the data may not be as complete as hospital registries; for example, mild injection site reactions may be overlooked and underreported by clinicians.

Musculoskeletal and connective tissue disorders were common in patients receiving alirocumab or evolocumab. The muscle-related AE signals were mainly manifested as back pain, myalgia, pain in extremity, arthralgia, and muscle spasms, which was consistent with evidence from previous clinical trials (Robinson et al., 2015; Moriarty et al., 2020). In addition, the post-marketing pharmacovigilance studies also suggested the relation between PCSK9 inhibitors and muscle symptoms in clinical practice (Gurgoze et al., 2019; Ding et al., 2022). It is well-known that muscle-related AEs were common side effects of statins. When compared to statins/ezetimibe, our results suggested that PCSK9 inhibitors were relatively safe. Nevertheless, our observations and previous real-world studies advocate the necessity to monitor for muscle symptoms in clinical practice (Gurgoze et al., 2019). Furthermore, since estrogen competes with statins for transporters and enzymes, and female gender has previously been deemed as a risk factor for musculoskeletal disorders (Faubion et al., 2019), PCSK9 inhibitors have the potential as a better alternative for women.

Additionally, PCSK9 inhibitors were associated with an increased risk of influenza like illness and infections, such as nasopharyngitis and influenza. This finding was also in line with previous studies (Ji et al., 2022; Santos et al., 2022). For example, a single-arm, open-label extension of HAUSER-RCT reported that the most common AEs associated with evolocumab were nasopharyngitis, headache and influenza-like illness (Santos et al., 2022). Influenza like illness was frequently reported in the Erasmus Medical Centre hospital (EMC) registry, Lareb and VigiLyza databases (Gurgoze et al., 2019). Influenza is common in the general population and is often overlooked by clinicians. However, these signals still need attention, especially in people below 65 years of age who reported a higher frequency of influenza like illness with alirocumab and evolocumab.

Evolocumab, but not alirocumab, had a significantly higher reporting risk of injury, poisoning and procedural complications. This difference may be related to the higher sales of evolocumab in the marketplace and the consequent higher frequency of AE reported. Unfortunately, AEs associated with this SOC are rarely reported in clinical trials and other non-mandatory spontaneous systems such as JADER (Nomura et al., 2015). Nevertheless, this finding suggests clinicians to pay special attention to these adverse outcomes when using evolocumab, especially in elderly patients.

This study did not fully support the causal relationship between PCSK9 inhibitors and cognitive impairment, which was in consistent with recent studies (Giugliano et al., 2017a; Guedeney et al., 2019). In addition, PCSK9 inhibitors had a lower reporting probability of nervous system and psychiatric disorders compared with various statins and ezetimibe, which were seldomly investigated previously. Despite the potential associations between lipid-lowering therapies (LLTs) and neurocognitive disorders remain an area of debate, our observed favorable effect of PCSK9 inhibitor on cognitive functions advocates its use in real-world settings when patients are at high risk of neurocognitive disorders. Nevertheless, since few studies have evaluated adverse cognitive effects associated with PCSK9 inhibitors in real-world contexts or compared them with statins, no firm conclusion can be drawn from our findings.

In this study, a positive diabetes signal was observed only with evolocumab (ROR = 2.5) and not with alirocumab. Although diabetes and worsening glycemic control were not found to be associated with PCSK9 inhibitors in clinical trials (Carvalho et al., 2017; Chiu et al., 2020), it has been suggested that the FOURIER trial may not be robust enough to detect diabetes risk (van Bruggen and Luijendijk, 2019). However, in the present study, the lower risk of metabolism and nutrition disorders of PCSK9 inhibitors compared to statins provide reassurance regarding their clinical benefit. Considering the possible diabetogenic effects of high-dose statins, PCSK9 inhibitors may be preferable for patients who need high-intensity lipid-lowering treatment but are at high risk of new-onset diabetes.

For some undocumented AEs, such as acute cardiac event and xanthoma, although the expected incidence was much lower than other AE signals in our study (0.004%–0.019%), clinicians should take them seriously. Since PCSK9 inhibitors have been shown to effectively reduce the risk of ASCVD and tendon xanthoma (Bea et al., 2017), the underlying mechanism and exact effects are waiting to be revealed.

Several limitations in this study should be acknowledged. Firstly, FAERS does not provide background information on the number of patients who took the drug. The number of AE reports depends greatly on the reporting behavior of individuals. Therefore, we can only roughly estimate the incidence of AEs by the signal strength (ROR value), but cannot directly calculate or rely on it to infer causality. Another practical problem is the insufficient reporting rate. However, with the popularity of PCSK9 inhibitors since 2015, there will be more reports on these drugs. Although FAERS itself is not an absolute indicator of drug safety, we believe that it is of great value in continuously monitoring the safety of PCSK9 inhibitors, which will help to better characterize their safety profile in the real-world context. Moreover, the accuracy of our findings heavily depends on the quantity and quality of the information entered into FAERS. However, some studies claimed that only 5% of serious AEs were literally submitted (Begaud et al., 2002); hence, severe adverse effects may be underestimated. In addition, as FAERS typically lacks information on concomitant and repeated use of drugs, we may overestimate or underestimate the association between the target drug and suspected AEs. Finally, due to the restriction of the data types collected by FAERS, we have not been able to compare the risk of PCSK9 inhibitors with different doses of statins, nor have we been able to evaluate their long-term safety.

## Conclusion

In conclusion, we investigated the safety profile of PCSK9 inhibitors based on real-world data from FAERS. Compared with all other drugs, PCSK9 inhibitors were associated with an increased reporting risk of musculoskeletal and connective tissue disorders and general disorders and administration site conditions, overall and by subgroups. The most notable AEs were injection site reactions and muscle toxicity. Compared with statins/ezetimibe, PCSK9 inhibitors exhibited a lower reporting probability of adverse events associated with “nervous system disorders”, “psychiatric disorders” and “metabolism and nutrition disorders”, but mixed results for musculoskeletal disorders. We also reported some undocumented AE signals. Further studies are still needed to establish causality and validate our results.

## Data Availability

The datasets presented in this study can be found in online repositories. The names of the repository/repositories and accession number(s) can be found below: https://fis.fda.gov/extensions/FPD-QDE-FAERS/FPD-QDE-FAERS.html.
