# Supplementary material for: Real-world safety of PCSK9 inhibitors: A pharmacovigilance study based on spontaneous reports in FAERS
Source: Front Pharmacol. 2022 Nov 24;13:894685. doi: 10.3389/fphar.2022.894685 (PMC9729267; doi:10.3389/fphar.2022.894685)
Supplement: Supplementary file 1 [file DataSheet1.docx]

Supplementary material

Table S1. Two-by-two contingency table for disproportionality analysis

|  | Target event | Other events | Sums |
| --- | --- | --- | --- |
| Target drug | a | b | a+b |
| Other drugs | c | d | c+d |
| Sums | a+c | b+d | a+b+c+d |

ROR=$\left( a/c \right)/\left( b/d \right)$; ROR 95%CI=$e^{\mathrm{In}\left( ROR \right)\pm1.96\sqrt{\left( \frac{1}{a}+\frac{1}{b}+\frac{1}{c}+\frac{1}{d} \right)}}$; PRR=$\frac{a/\left( a+b \right)}{c/\left( c+d \right)}$; $\chi^{2}=\frac{\left( \left| ad-bc \right|-N/2 \right)^{2}N}{\left( a+b \right)\left( a+c \right)\left( c+d \right)\left( b+d \right)}$

Table S2. Top 5 AEs of alirocumab by subgroups

| AE signals | No. of reports (%) |
| --- | --- |
| ***Female*** |  |
| Myalgia | 675 (8.1) |
| Muscle spasms | 501 (6.0) |
| Injection site pain | 475 (5.7) |
| Arthralgia | 431 (5.2) |
| Pain in extremity | 420 (5.1) |
| ***Male*** |  |
| Myalgia | 525 (10.3) |
| Arthralgia | 289 (5.6) |
| Muscle spasms | 287 (5.6) |
| Influenza like illness | 264 (5.2) |
| Pain in extremity | 229 (4.5) |
| ***<65 years*** |  |
| Myalgia | 351 (8.8) |
| Arthralgia | 233 (5.9) |
| Influenza like illness | 221 (5.6) |
| Injection site pain | 208 (5.2) |
| Muscle spasms | 192 (4.8) |
| ***≥65 years*** |  |
| Myalgia | 569 (8.4) |
| Muscle spasms | 425 (6.2) |
| Pain in extremity | 367 (5.4) |
| Arthralgia | 356 (5.2) |
| Injection site pain | 328 (4.8) |

Table S3. Top 5 AEs of evolocumab by subgroups

| AE signals | No. of reports (%) |
| --- | --- |
| ***Female*** |  |
| Injection site pain | 4413 (13.6) |
| Back pain | 2260 (7.0) |
| Injection site bruising | 2165 (6.7) |
| Myalgia | 1991 (6.1) |
| Rhinorrhoea | 1663 (5.1) |
| ***Male*** |  |
| Injection site pain | 2081 (10.0) |
| Back pain | 1719 (8.3) |
| Myalgia | 1669 (8.0) |
| Rhinorrhoea | 1180 (5.7) |
| Arthralgia | 1172 (5.6) |
| ***<65 years*** |  |
| Injection site pain | 2169 (12.9) |
| Back pain | 1263 (7.5) |
| Injection site bruising | 1089 (6.5) |
| Myalgia | 1047 (6.2) |
| Influenza like illness | 910 (5.4) |
| ***≥65 years*** |  |
| Injection site pain | 2686 (11.3) |
| Back pain | 1821 (7.7) |
| Myalgia | 1589 (6.7) |
| Rhinorrhoea | 1393 (5.9) |
| Injection site bruising | 1319 (5.6) |

Table S4. AE signals of alirocumab at the system organ class (SOC) and preferred terms level

| AE signals | No. of reports | ROR (95% CI) | Chi-square value |
| --- | --- | --- | --- |
| ***Cardiac disorders*** | | | |
| Myocardial infarction | 151 | 2.1 (1.8-2.4) | 83.2 |
| Angina pectoris | 40 | 2.4 (1.7-3.2) | 31.6 |
| Cardiac flutter | 12 | 2.8 (1.6-5.0) | 14.2 |
| Angina unstable | 11 | 3.9 (2.1-7.0) | 23.1 |
| Acute cardiac event | 3 | 30 (9.4-95.3) | 80.7 |
| Cardiac disorder | 3 | 3.6 (1.2-11.3) | 5.7 |
| Aortic valve disease | 3 | 3.3 (1.1-10.3) | 4.8 |
| ***Congenital, familial and genetic disorders*** | | | |
| Type IIa hyperlipidaemia | 3 | 15.6 (5.0-48.8) | 40.0 |
| Muscular dystrophy | 3 | 12.3 (3.9-38.6) | 30.7 |
| ***Ear and labyrinth disorders*** | | | |
| Blepharospasm | 9 | 3.2 (1.7-6.1) | 13.5 |
| Middle ear effusion | 5 | 2.9 (1.2-7.0) | 6.3 |
| Ear pruritus | 5 | 2.5 (1.0-6.0) | 4.4 |
| Inner ear disorder | 3 | 4.3 (1.4-13.3) | 7.5 |
| ***Gastrointestinal disorders*** | | | |
| Abnormal faeces | 14 | 2.2 (1.3-3.8) | 9.5 |
| Mouth swelling | 11 | 2.2 (1.2-4) | 7.2 |
| Sensitivity of teeth | 4 | 3.2 (1.2-8.7) | 6.2 |
| Oral pruritus | 4 | 2.9 (1.1-7.7) | 4.9 |
| ***General disorders and administration site conditions*** | | | |
| Injection site pain | 732 | 4.6 (4.2-4.9) | 1976.1 |
| Influenza like illness | 645 | 11.9 (11-12.9) | 6240.4 |
| Injection site bruising | 558 | 10.7 (9.9-11.7) | 4786.1 |
| Injection site erythema | 483 | 7.8 (7.1-8.5) | 2785.2 |
| Injection site haemorrhage | 372 | 8.1 (7.3-9.0) | 2268.6 |
| Injection site swelling | 307 | 8.1 (7.2-9.0) | 1861.4 |
| Injection site pruritus | 293 | 8.4 (7.5-9.5) | 1883.2 |
| Injection site reaction | 192 | 4.8 (4.1-5.5) | 563.0 |
| Injection site mass | 186 | 6.8 (5.8-7.8) | 897.6 |
| Injection site rash | 185 | 11.8 (10.2-13.7) | 1800.0 |
| Therapeutic response decreased | 130 | 3.5 (3.0-4.2) | 235.6 |
| Injection site discolouration | 93 | 11.9 (9.7-14.7) | 914.6 |
| Injection site warmth | 77 | 7.8 (6.3-9.8) | 454.6 |
| Injection site urticaria | 66 | 5.3 (4.2-6.8) | 230.6 |
| Injection site induration | 47 | 6.3 (4.7-8.4) | 207.4 |
| Secretion discharge | 39 | 3.8 (2.8-5.2) | 80.3 |
| Injection site extravasation | 29 | 2.9 (2-4.1) | 35.0 |
| Injection site haematoma | 26 | 12.1 (8.2-17.8) | 259.6 |
| Injection site irritation | 26 | 7.1 (4.8-10.5) | 135.1 |
| Injection site discomfort | 21 | 3.1 (2-4.7) | 28.9 |
| Injection site inflammation | 17 | 5.8 (3.6-9.4) | 67.4 |
| Injection site vesicles | 17 | 5.2 (3.2-8.4) | 57.4 |
| Injection site injury | 16 | 5.9 (3.6-9.6) | 64.4 |
| Injection site hypoaesthesia | 15 | 14 (8.4-23.3) | 177.3 |
| Application site injury | 14 | 87.3 (50.1-152) | 1062.9 |
| Injection site indentation | 12 | 11.2 (6.3-19.8) | 109.8 |
| Injection site exfoliation | 11 | 29.2 (16-53.3) | 287.3 |
| Injection site hypersensitivity | 10 | 5.2 (2.8-9.7) | 33.6 |
| Injection site dryness | 9 | 18.7 (9.6-36.2) | 146.9 |
| Injection site scar | 9 | 4.9 (2.5-9.5) | 27.8 |
| Administration site bruise | 7 | 25.1 (11.8-53.4) | 156.5 |
| Administration site indentation | 6 | 185.3 (75.5-455.2) | 872.3 |
| Injection site scab | 4 | 6.2 (2.3-16.5) | 17.1 |
| Administration site pain | 4 | 5.6 (2.1-15) | 15.0 |
| Injection site paraesthesia | 4 | 4.4 (1.6-11.8) | 10.5 |
| Skin induration | 4 | 3.5 (1.3-9.4) | 7.2 |
| Injection site eczema | 3 | 33.8 (10.6-107.7) | 91.2 |
| Injection site streaking | 3 | 22.2 (7.0-70.0) | 58.9 |
| ***Infections and infectations*** | | | |
| Nasopharyngitis | 381 | 2.6 (2.4-2.9) | 373.3 |
| Influenza | 223 | 2.4 (2.1-2.8) | 183.8 |
| Viral upper respiratory tract infection | 19 | 2.9 (1.8-4.5) | 22.9 |
| Rhinitis | 18 | 2.9 (1.8-4.6) | 22.2 |
| Laryngitis | 16 | 2.1 (1.3-3.5) | 9.6 |
| ***Metabolism and nutrition disorders*** | | | |
| Gout | 26 | 2.1 (1.4-3.0) | 14.5 |
| ***Musculoskeletal and connective tissue disorders*** | | | |
| Myalgia | 1246 | 11.5 (10.8-12.1) | 11359.4 |
| Muscle spasms | 821 | 6.5 (6.0-6.9) | 3694.8 |
| Arthralgia | 753 | 2.7 (2.5-2.9) | 784.5 |
| Pain in extremity | 686 | 3.1 (2.9-3.4) | 988.3 |
| Back pain | 360 | 2.1 (1.9-2.4) | 215.2 |
| Muscular weakness | 233 | 3.1 (2.7-3.5) | 321.2 |
| Musculoskeletal pain | 162 | 3.4 (2.9-3.9) | 266.4 |
| Bone pain | 89 | 2.0 (1.6-2.5) | 45.0 |
| Neck pain | 81 | 2.0 (1.6-2.5) | 41.9 |
| Limb discomfort | 67 | 2.4 (1.9-3.1) | 56.4 |
| Musculoskeletal discomfort | 60 | 4.5 (3.5-5.7) | 159.6 |
| Joint stiffness | 47 | 2.5 (1.9-3.3) | 41.3 |
| Muscle tightness | 37 | 3.3 (2.4-4.6) | 59.2 |
| Muscle disorder | 33 | 6.5 (4.6-9.1) | 151.0 |
| Back disorder | 30 | 2.5 (1.8-3.6) | 27.9 |
| Muscle fatigue | 24 | 8.1 (5.4-12.2) | 148.2 |
| Muscle atrophy | 17 | 2.4 (1.5-3.9) | 13.7 |
| Tendon pain | 11 | 2.1 (1.1-3.7) | 6.0 |
| Muscle rupture | 8 | 2.9 (1.5-5.9) | 10.2 |
| Polymyalgia rheumatica | 6 | 3.6 (1.6-7.9) | 11.0 |
| Tendon discomfort | 5 | 7.6 (3.1-18.2) | 28.1 |
| Muscle discomfort | 3 | 7.8 (2.5-24.4) | 17.6 |
| Ligament disorder | 3 | 5.0 (1.6-15.7) | 9.6 |
| ***Neoplasms benign, malignant and unspecified*** | | | |
| Transitional cell carcinoma | 5 | 6.6 (2.7-15.9) | 23.4 |
| Bladder transitional cell carcinoma | 4 | 3.1 (1.1-8.2) | 5.6 |
| ***Nervous system disorders*** | | | |
| Amnesia | 91 | 2.2 (1.8-2.6) | 56.4 |
| Head discomfort | 31 | 2.2 (1.5-3.1) | 19.4 |
| Hypokinesia | 27 | 2.3 (1.6-3.3) | 19.3 |
| Sinus headache | 17 | 5.0 (3.1-8.0) | 53.5 |
| Carotid artery disease | 3 | 4.8 (1.6-15.1) | 9.1 |
| ***Psychiatric disorders*** | | | |
| Middle insomnia | 27 | 2.2 (1.5-3.3) | 18.4 |
| ***Renal and urinary disorders*** |  |  |  |
| Chromaturia | 33 | 2.2 (1.6-3.1) | 21.8 |
| Urine odour abnormal | 14 | 3.2 (1.9-5.5) | 21.8 |
| Bladder pain | 8 | 3.6 (1.8-7.3) | 15.3 |
| Urinary bladder polyp | 4 | 20.3 (7.5-54.8) | 71.3 |
| Urinary tract discomfort | 3 | 9.8 (3.1-30.6) | 23.3 |
| Bladder discomfort | 3 | 3.3 (1.1-10.2) | 4.8 |
| ***Reproductive system and breast disorders*** | | | |
| Breast disorder | 5 | 4 (1.7-9.7) | 11.2 |
| Nipple pain | 4 | 3.3 (1.2-8.7) | 6.2 |
| Genital rash | 3 | 3.3 (1.1-10.3) | 4.8 |
| ***Respiratory, thoracic and mediastinal disorders*** | | | |
| Cough | 469 | 2.3 (2.1-2.5) | 349.6 |
| Rhinorrhoea | 436 | 8.8 (8-9.7) | 2939.4 |
| Oropharyngeal pain | 249 | 3.6 (3.2-4.1) | 459.8 |
| Nasal congestion | 157 | 3.6 (3.1-4.2) | 295.7 |
| Dysphonia | 92 | 2.2 (1.8-2.7) | 62.0 |
| Sinus disorder | 75 | 5 (4-6.3) | 236.8 |
| Throat irritation | 67 | 2.3 (1.8-3) | 51.4 |
| Sneezing | 58 | 3.9 (3-5.1) | 126.0 |
| Sinus congestion | 38 | 3.9 (2.8-5.4) | 81.8 |
| Respiratory tract congestion | 30 | 2.7 (1.9-3.8) | 30.9 |
| Upper-airway cough syndrome | 22 | 3.5 (2.3-5.2) | 38.0 |
| Pharyngeal oedema | 22 | 2.1 (1.4-3.3) | 13.5 |
| Oropharyngeal discomfort | 16 | 3.1 (1.9-5) | 22.4 |
| Paranasal sinus discomfort | 14 | 4.5 (2.7-7.7) | 38.2 |
| Nasal discomfort | 14 | 2.5 (1.5-4.3) | 13.0 |
| Paranasal sinus hypersecretion | 13 | 4.9 (2.8-8.4) | 40.0 |
| Choking sensation | 10 | 2.6 (1.4-4.9) | 10.0 |
| Throat clearing | 9 | 5 (2.6-9.6) | 28.3 |
| Nasal disorder | 8 | 3.8 (1.9-7.6) | 16.2 |
| Sinus pain | 8 | 3.6 (1.8-7.3) | 15.2 |
| Upper respiratory tract congestion | 7 | 3.5 (1.6-7.3) | 12.2 |
| Respiratory symptom | 6 | 3.0 (1.3-6.6) | 7.7 |
| Respiratory tract irritation | 3 | 4.3 (1.4-13.4) | 7.6 |
| Nasal inflammation | 3 | 3.6 (1.2-11.3) | 5.7 |
| ***Skin and subcutaneous tissue disorders*** | | | |
| Pruritus | 512 | 2.3 (2.1-2.5) | 371.2 |
| Pruritus generalised | 81 | 4.7 (3.8-5.9) | 235.0 |
| Rash pruritic | 81 | 2.5 (2-3.1) | 71.8 |
| Rash generalised | 58 | 3.2 (2.5-4.1) | 86.7 |
| Skin haemorrhage | 21 | 2.4 (1.5-3.6) | 16.2 |
| Skin warm | 13 | 3.3 (1.9-5.7) | 20.7 |
| Pigmentation disorder | 13 | 3.0 (1.7-5.2) | 17.2 |
| Skin mass | 13 | 2.5 (1.4-4.3) | 11.5 |
| Skin atrophy | 11 | 2.6 (1.4-4.7) | 10.6 |
| Spider vein | 4 | 9.3 (3.5-25.1) | 29.4 |
| ***Vascular disorders*** | | | |
| Angiopathy | 17 | 5.5 (3.4-8.9) | 62.5 |
| Arterial occlusive disease | 17 | 3.7 (2.3-5.9) | 33.0 |
| Vascular occlusion | 9 | 6.7 (3.5-12.8) | 43.0 |
| Varicose vein | 8 | 2.1 (1.1-4.3) | 4.9 |
| Peripheral artery occlusion | 6 | 4.0 (1.8-8.9) | 13.3 |

Table S5. AE signals of evolocumab at the system organ class (SOC) and preferred terms level

| AE signals | No. of reports | ROR (95% CI) | Chi-square value |
| --- | --- | --- | --- |
| ***Blood and lymphatic system disorders*** | | | |
| Increased tendency to bruise | 98 | 3.5 (2.9-4.3) | 174.1 |
| Lymph node pain | 10 | 2.2 (1.2-4) | 6.2 |
| ***Cardiac disorders*** | | | |
| Coronary artery occlusion | 62 | 2.2 (1.7-2.9) | 41 |
| Coronary artery stenosis | 22 | 2.6 (1.7-3.9) | 20.8 |
| ***Congenital, familial and genetic disorders*** | | | |
| Type IIa hyperlipidaemia | 3 | 3.9 (1.2-12.2) | 6.3 |
| Muscular dystrophy | 6 | 6.3 (2.8-14.1) | 25.6 |
| ***Ear and labyrinth disorders*** | | | |
| Hypoacusis | 357 | 2.5 (2.3-2.8) | 321.4 |
| Ear congestion | 30 | 4.1 (2.9-5.9) | 69.6 |
| Middle ear effusion | 16 | 2.4 (1.4-3.9) | 12.3 |
| Eustachian tube disorder | 3 | 4.4 (1.4-13.8) | 7.7 |
| ***Gastrointestinal disorders*** | | | |
| Sensitivity of teeth | 11 | 2.2 (1.2-4.1) | 7.5 |
| Lip pruritus | 7 | 3.4 (1.6-7.2) | 11.6 |
| Tongue exfoliation | 3 | 3.2 (1-10.2) | 4.6 |
| ***General disorders and administration site conditions*** | | | |
| Injection site pain | 6541 | 10.9 (10.7-11.2) | 53507.3 |
| Injection site bruising | 3225 | 16.8 (16.2-17.4) | 42972.3 |
| Influenza like illness | 2397 | 11.6 (11.1-12.1) | 21419.8 |
| Injection site haemorrhage | 2017 | 11.6 (11.1-12.1) | 18109.6 |
| Injection site swelling | 1277 | 8.7 (8.2-9.2) | 8213.7 |
| Injection site erythema | 1138 | 4.6 (4.3-4.9) | 3124.3 |
| Injection site mass | 827 | 7.8 (7.2-8.3) | 4636.4 |
| Injection site pruritus | 534 | 3.9 (3.5-4.2) | 1104.9 |
| Unevaluable event | 507 | 2.3 (2.1-2.5) | 353.5 |
| Therapeutic response unexpected | 394 | 2.5 (2.3-2.8) | 364.8 |
| Injection site discomfort | 368 | 14.4 (13-16) | 4244.9 |
| Injection site reaction | 339 | 2.1 (1.9-2.3) | 193.5 |
| Injection site discolouration | 303 | 10.1 (9-11.3) | 2345.7 |
| Therapy non-responder | 284 | 2.5 (2.2-2.8) | 239 |
| Injection site rash | 282 | 4.5 (4-5.1) | 758 |
| Injection site urticaria | 272 | 5.6 (5-6.3) | 1002.1 |
| Injection site induration | 179 | 6.2 (5.3-7.1) | 744.7 |
| Injection site extravasation | 175 | 4.4 (3.8-5.1) | 450 |
| Injection site vesicles | 142 | 11.5 (9.7-13.6) | 1277.7 |
| Injection site warmth | 121 | 3.1 (2.6-3.7) | 169 |
| Injection site irritation | 96 | 6.7 (5.5-8.3) | 451.9 |
| Secretion discharge | 84 | 2.1 (1.7-2.6) | 45.4 |
| Sluggishness | 73 | 2.4 (1.9-3.1) | 61.3 |
| Injection site haematoma | 54 | 6.4 (4.9-8.4) | 236.6 |
| Injection site injury | 49 | 4.6 (3.5-6.1) | 133.9 |
| Application site swelling | 49 | 3.8 (2.9-5) | 98.5 |
| Application site haemorrhage | 44 | 3.7 (2.8-5) | 86 |
| Injection site inflammation | 36 | 3.1 (2.2-4.3) | 50.7 |
| Injection site paraesthesia | 34 | 9.8 (7-13.9) | 255 |
| Injection site indentation | 33 | 7.9 (5.6-11.2) | 190.9 |
| Injection site hypersensitivity | 33 | 4.4 (3.1-6.2) | 83.3 |
| Injection site hypoaesthesia | 31 | 7.4 (5.2-10.6) | 164 |
| Application site mass | 26 | 20.1 (13.4-30.2) | 425 |
| Injection site scar | 19 | 2.6 (1.7-4.1) | 18.6 |
| Injection site papule | 18 | 2 (1.3-3.2) | 9.1 |
| Injection site laceration | 16 | 10 (6.1-16.6) | 123.2 |
| Application site discomfort | 15 | 2.3 (1.4-3.8) | 10.7 |
| Injection site macule | 13 | 7.4 (4.3-12.9) | 69.3 |
| Injection site scab | 13 | 5.1 (2.9-8.8) | 41.6 |
| Application site urticaria | 13 | 2.1 (1.2-3.7) | 7.7 |
| Puncture site pain | 12 | 28 (15.2-51.5) | 270.1 |
| Application site bruise | 11 | 3 (1.6-5.4) | 14.4 |
| Puncture site haemorrhage | 10 | 5.2 (2.8-9.8) | 33.1 |
| Injection site hyperaesthesia | 9 | 12.5 (6.3-24.5) | 88.8 |
| Injection site oedema | 9 | 2.4 (1.3-4.7) | 7.5 |
| Injection site coldness | 7 | 6 (2.8-12.8) | 28.4 |
| Oedema mucosal | 7 | 3.2 (1.5-6.7) | 10.2 |
| Vascular stent occlusion | 6 | 3.3 (1.5-7.4) | 9.4 |
| Injection site exfoliation | 5 | 3.2 (1.3-7.8) | 7.6 |
| Puncture site swelling | 4 | 44.4 (14.8-132.7) | 135.7 |
| Injection site streaking | 3 | 5.6 (1.8-17.5) | 10.8 |
| Injection site thrombosis | 3 | 4.8 (1.5-15.2) | 8.9 |
| Drug effect faster than expected | 3 | 3.5 (1.1-11.1) | 5.3 |
| ***Hepatobiliary disorders*** | | | |
| Non-alcoholic fatty liver | 6 | 7 (3.1-15.8) | 29.7 |
| ***Infections and infectations*** | | | |
| Nasopharyngitis | 1816 | 3.2 (3-3.3) | 2600.6 |
| Influenza | 871 | 2.4 (2.2-2.5) | 686.1 |
| Upper respiratory tract infection | 302 | 2.1 (1.9-2.4) | 179.2 |
| Laryngitis | 88 | 3 (2.4-3.7) | 113.4 |
| Rhinitis | 54 | 2.2 (1.7-2.9) | 34.3 |
| Viral pharyngitis | 5 | 3.8 (1.6-9.1) | 9.9 |
| ***Metabolism and nutrition disorders*** | | | |
| Glucose tolerance impaired | 35 | 2.5 (1.8-3.5) | 30.5 |
| Weight loss poor | 20 | 2.5 (1.6-4) | 18.6 |
| ***Musculoskeletal and connective tissue disorders*** | | | |
| Back pain | 4008 | 6.2 (6-6.4) | 16595.9 |
| Myalgia | 3687 | 8.7 (8.4-9) | 23352.3 |
| Arthralgia | 2669 | 2.4 (2.3-2.5) | 2124.7 |
| Pain in extremity | 2441 | 2.8 (2.7-3) | 2804.4 |
| Muscle spasms | 2255 | 4.5 (4.3-4.7) | 5883.7 |
| Muscular weakness | 738 | 2.5 (2.3-2.6) | 620 |
| Musculoskeletal pain | 641 | 3.4 (3.1-3.6) | 1044.5 |
| Mobility decreased | 583 | 2.8 (2.5-3) | 643.2 |
| Neck pain | 368 | 2.3 (2.1-2.6) | 273.3 |
| Musculoskeletal disorder | 188 | 3.1 (2.6-3.5) | 256 |
| Muscle disorder | 151 | 7.6 (6.5-9) | 834.9 |
| Musculoskeletal discomfort | 140 | 2.6 (2.2-3.1) | 137.8 |
| Muscle tightness | 133 | 3 (2.5-3.6) | 174.8 |
| Back disorder | 127 | 2.7 (2.3-3.2) | 135.6 |
| Groin pain | 64 | 2.4 (1.9-3.1) | 52.7 |
| Muscle fatigue | 38 | 3.2 (2.4-4.5) | 57.6 |
| Joint lock | 23 | 2.8 (1.9-4.2) | 26.3 |
| Muscle injury | 23 | 2.2 (1.5-3.4) | 15.5 |
| Polymyalgia rheumatica | 20 | 3 (1.9-4.7) | 26.1 |
| Joint noise | 13 | 2.1 (1.2-3.7) | 7.9 |
| Muscle mass | 7 | 4.9 (2.3-10.4) | 21.1 |
| Ligament pain | 6 | 3.1 (1.4-7) | 8.5 |
| Bursa disorder | 3 | 3.6 (1.1-11.2) | 5.4 |
| ***Nervous system disorders*** | | | |
| Sleep disorder due to a general medical condition | 177 | 5.9 (5-6.8) | 691.5 |
| Sleep disorder due to general medical condition, insomnia type | 61 | 6.2 (4.8-8) | 256.3 |
| Sinus headache | 39 | 2.9 (2.1-4) | 46.9 |
| Carotid artery occlusion | 22 | 3 (2-4.6) | 28.8 |
| Carotid artery stenosis | 13 | 2.2 (1.3-3.8) | 8.2 |
| Chronic inflammatory demyelinating polyradiculoneuropathy | 8 | 2.4 (1.2-4.9) | 6.7 |
| Carotid arteriosclerosis | 6 | 2.3 (1-5.1) | 4.3 |
| Phantom pain | 3 | 3.9 (1.3-12.4) | 6.4 |
| ***Psychiatric disorders*** | | | |
| Fear of injection | 348 | 19.7 (17.6-22) | 5556 |
| ***Renal and urinary disorders*** | | | |
| Urinary tract discomfort | 5 | 4.1 (1.7-10) | 11.5 |
| ***Respiratory, thoracic and mediastinal disorders*** | | | |
| Rhinorrhoea | 2861 | 15.6 (15-16.2) | 35269.3 |
| Oropharyngeal pain | 1137 | 4.2 (3.9-4.4) | 2661.6 |
| Nasal congestion | 511 | 3 (2.7-3.2) | 658.9 |
| Dysphonia | 384 | 2.4 (2.1-2.6) | 292.6 |
| Sinus disorder | 303 | 5.1 (4.6-5.8) | 980.5 |
| Throat irritation | 256 | 2.3 (2-2.5) | 176.9 |
| Sneezing | 235 | 4 (3.6-4.6) | 526.9 |
| Respiratory tract congestion | 197 | 4.5 (3.9-5.1) | 515.1 |
| Upper-airway cough syndrome | 149 | 6 (5.1-7.1) | 600.2 |
| Paranasal sinus hypersecretion | 113 | 11.2 (9.3-13.6) | 989.8 |
| Sinus congestion | 107 | 2.8 (2.3-3.4) | 119.9 |
| Aphonia | 97 | 2.4 (2-3) | 81.4 |
| Dry throat | 76 | 2.7 (2.2-3.4) | 82.4 |
| Respiratory symptom | 29 | 3.6 (2.5-5.2) | 53.9 |
| Nasal disorder | 20 | 2.4 (1.5-3.7) | 15.7 |
| Pharyngeal disorder | 19 | 2.4 (1.5-3.8) | 15 |
| Upper respiratory tract congestion | 19 | 2.4 (1.5-3.7) | 14.8 |
| Sinus pain | 18 | 2.1 (1.3-3.3) | 9.7 |
| Throat clearing | 16 | 2.2 (1.4-3.6) | 10.6 |
| Pharyngeal erythema | 14 | 2.5 (1.5-4.2) | 12.4 |
| Nasal pruritus | 12 | 2.9 (1.6-5.1) | 14.5 |
| Allergic sinusitis | 8 | 3.4 (1.7-6.8) | 13 |
| Pharyngeal hypoaesthesia | 6 | 3 (1.3-6.7) | 7.9 |
| Nasal crusting | 6 | 2.6 (1.1-5.8) | 5.8 |
| Increased viscosity of upper respiratory secretion | 6 | 2.3 (1-5.2) | 4.5 |
| Sinonasal obstruction | 4 | 3.5 (1.3-9.3) | 6.9 |
| ***Skin and subcutaneous tissue disorders*** | | | |
| Rash pruritic | 403 | 3.1 (2.8-3.5) | 575.9 |
| Pruritus generalised | 220 | 3.2 (2.8-3.7) | 333 |
| Rash generalised | 162 | 2.2 (1.9-2.6) | 110.1 |
| Papule | 52 | 2.9 (2.2-3.8) | 63.5 |
| Rash vesicular | 34 | 3 (2.1-4.2) | 43.4 |
| Skin odour abnormal | 21 | 2 (1.3-3.1) | 11 |
| Exfoliative rash | 14 | 2.4 (1.4-4.1) | 11.3 |
| Nail growth abnormal | 10 | 2.8 (1.5-5.2) | 11.1 |
| Spider vein | 5 | 2.9 (1.2-7.1) | 6.2 |
| Xanthoma | 4 | 9.3 (3.4-25.5) | 28.3 |
| ***Vascular disorders*** | | | |
| Vascular occlusion | 19 | 3.5 (2.3-5.6) | 34.2 |
| Intermittent claudication | 15 | 3.7 (2.2-6.1) | 28.4 |
| Peripheral artery occlusion | 14 | 2.3 (1.4-4) | 10.6 |
| Arterial disorder | 11 | 2.7 (1.5-4.9) | 11.7 |
| Vascular pain | 9 | 2.8 (1.4-5.4) | 10.1 |
| Vascular calcification | 5 | 2.6 (1.1-6.3) | 4.8 |
